# Paternity After Treatment of Cryptorchidism: A Systematic Review

**DOI:** 10.3390/jcm14134768

**Published:** 2025-07-05

**Authors:** Anna Lund Henriksen, Ida-Marie Poulsen, Freja Sørensen, Jens Fedder

**Affiliations:** 1 Centre of Andrology & Fertility Clinic, Department D, Odense University Hospital, DK-5000 Odense, Denmark; a.l.henriksen@me.com (A.L.H.); idamarie_poulsen@hotmail.com (I.-M.P.); frejarikke123@hotmail.com (F.S.); 2 Department of Clinical Medicine, University of Southern Denmark, DK-5000 Odense, Denmark; 3 Centre of Andrology & Fertility Clinic, Horsens Hospital, DK-8700 Horsens, Denmark

**Keywords:** undescended testis, cryptorchidism, orchiopexy, treatment, paternity

## Abstract

**Background:** Male infertility can arise from various causes, accounting for 30–50% of infertility cases. The aim of this systematic review is to establish paternity outcomes in men treated for cryptorchidism during childhood, and to evaluate the optimal age for surgical intervention in relation to fertility. **Methods:** This systematic review is conducted according to the PRISMA guidelines and registered in PROSPERO (CRD420251010710). The electronic databases Medline, Embase, and PubMed were searched for eligible studies from 1990 to February 2025. All types of original published human studies examining paternity outcomes in men treated for cryptorchidism during childhood were included. This review focused on comparing paternity rates between men treated for unilateral versus bilateral cryptorchidism. Additionally, studies were required to assess the influence of age at the time of treatment on the likelihood of achieving paternity later in life. Risk of bias was assessed using the Newcastle–Ottawa Scale. Six studies were included. **Results:** Five out of six studies found higher paternity rates in men treated for unilateral cryptorchidism compared to bilateral cryptorchidism. Early intervention is preferable, although delayed treatment in early childhood may still preserve fertility. One large study showed a 5% increase in the need for assisted reproductive treatment (ART) for every six-month delay in surgery, with a significantly higher use of ART observed when surgery was performed after 18 months. **Conclusions:** Bilateral cryptorchidism and delayed orchiopexy are linked to lower fertility and the increased use of ART. Future studies should focus on high-quality research to define the optimal age for orchiopexy in relation to paternity.

## 1. Introduction

Infertility is defined as the inability of couples to conceive after 12 months of trying. It is estimated that approximately 24% of couples have experienced infertility at some point in their lives [1]. Male factor infertility accounts for 30–50% of these cases. Infertility or subfertility can result from various causes, including testicular dysfunction, hormonal imbalances, lifestyle factors, and congenital abnormalities, including undescended testis (UDT) [2].

Undescended testis and cryptorchidism are terms that describe a testis that is not normally located at the bottom of the scrotum [3]. An undescended testis or cryptorchid testis refers to a testis that may either be located along its normal route of descent or found in an ectopic position, located outside the usual pathway in an abnormal location [4].

The prevalence of cryptorchidism has significantly increased over the past 40 years in boys with a normal birth weight [5]. The prevalence of cryptorchidism is around 2.1% of children born at term and 17.2% of children born preterm [6]. In Denmark, the prevalence is about 9% at birth, and after three months the prevalence decreases to 1.9% due to spontaneous descent [5]. If the testis remains cryptorchid beyond 6 months, orchiopexy is recommended to be carried out as soon as possible, preferably before the child reaches one year of age, to optimize fertility outcomes [7].

One of the long-term consequences of cryptorchidism is impaired semen quality and reduced fertility. In particular, men with a history of bilateral cryptorchidism show reduced sperm quality compared to those with a history of unilateral cryptorchidism. Furthermore, azoospermia is more frequently observed in men with bilateral cryptorchidism [8]. Fertility can either be assessed by paternity or by the results of semen and hormone analyses [9]. However, paternity is considered a more clinically relevant outcome, as a low sperm count does not always correlate with no paternity [10]. Fertility potential following an undescended testis has been a subject of ongoing debate. Early surgical treatment, ideally before one year of age, is thought to improve fertility outcomes, but its long-term effects on paternity remain unclear [7]. The aim of this study was to determine the chances of paternity after treatment for cryptorchidism. Specifically, we investigated the differences in paternity between men treated for unilateral and bilateral cryptorchidism, as well as the optimal age for surgical intervention in boys. To our knowledge, no systematic review has exclusively examined studies published after 1990 that compare paternity rates following unilateral versus bilateral cryptorchidism.

## 2. Materials and Methods

This systematic review was conducted and reported in accordance with Preferred Reporting Items for Systematic Reviews and Meta-Analysis 2020 (PRISMA 2020) guidelines [11] and was registered to PROSPERO, on 14 March 2025, and accepted on 18 March 2025. The registration number is CRD420251010710. 

### 2.1. Eligibility Criteria 

A study was found to be eligible for inclusion if the following criteria were met:

- *Population*: Men diagnosed with cryptorchidism during childhood.
- *Exposure*: Treatment for cryptorchidism before reaching adulthood.
- *Outcome*: The primary outcome was to determine the chances of paternity among men treated for unilateral versus bilateral cryptorchidism. Secondly, the study should assess how age at the time of treatment for cryptorchidism affects the likelihood of achieving paternity later in life, and the use of ART among men treated for cryptorchidism.

All types of original published human studies were included, while expert opinions, protocols, and studies under peer review were excluded. Publication time was limited from 1990 to the present due to methodological and clinical limitations, and the publication language was limited to English and Scandinavian languages to ensure accurate interpretation.

### 2.2. Information Sources and Search Strategy

The search strategy for Embase was conducted in collaboration with a research librarian from the University of Southern Denmark. We used the following search terms to search Embase: Cryptorchism; Cryptochi*; Undescended test*; Maldescensus testis; Testis, undescended; orchidopexy; Orchidopexi*; Orchiopex; surgery; Gonadorelin; gonadotropin releasing*; gonadorelin (Multi-purpose); GnRH; hCG; Surger*; Paternity; Fertility; Sperm Quality; Paternit*; Fertilit*; Sperm quality; Semen quality; Live birth; and Live Birth (Multi-purpose).

For the search in Embase, we used Emtree terms indicated by “/” and multi-purpose terms using “.mp”. The multi-purpose function in Embase covers the following fields: title, abstract, heading word, drug trade name, original title, device manufacturer, device trade name, keyword heading word, floating subheading word, and candidate term word. 

Some of the terms were truncated by “*” at the root to capture all variations and suffixes. 

Emtree terms in Embase were mapped to useful MeSH terms in Medline and PubMed. We conducted the search using the “.mp” (muti-purpose) function in Medline and the “All Fields” function in PubMed. The full search strategy is shown in Appendix A.

All of the search strategies were reviewed and approved by J.F. The last search run was on the 7 February 2025. Additional articles were manually retrieved through citation searching after reviewing the reference lists from relevant publications.

### 2.3. Study Selection and Data Collection

The eligibility assessment was performed independently in an unblinded standardized manner by two reviewers (A.L.H. and I.P.). All potentially eligible studies were screened based on their titles and abstracts, and potentially eligible studies were subsequently full-text-screened for inclusion. Disagreements between the two reviewers (A.L.H. and I.P.) were resolved by discussion internally; if no agreement could be reached, a third reviewer (F.S.) would decide. Covidence.org (accessed on 8 February 2025) was used as the screening and data extraction tool. In Covidence, a data extraction template was developed and pilot tested on one of the selected studies, and refined accordingly. One review author (A.L.H.) extracted data from the included studies and the second author (I.P.) systematically cross-checked all the data.

### 2.4. Data Items 

Information was extracted from each included study, regarding (1) study characteristics, including study design, sample size, the number of participants with an undescended testis, the number of controls, age groups, age at treatment, and the number of participants with unilateral as well as bilateral cryptorchidism; (2) outcome measures, including the number of men attempting paternity, the number and percentage of men who achieved paternity, the number and percentage of men who did not archive paternity, the use of assisted reproductive technology (ART), and other measured outcomes. 

### 2.5. Risk of Bias Assessment 

To assess the quality of the included records, two investigators (I.P. and A.L.H) independently evaluated the quality of the studies by using the Newcastle–Ottawa Scale (NOS). The NOS is designed to assess the quality of non-randomized studies by evaluating the selection of study groups, the comparability of the groups, and the ascertainment of exposure or outcomes, depending on whether the study is a case–control or cohort study. In this systematic review, the NOS for cohort studies was solely used to assess the validity of the included studies, with a maximum score of 9 stars [12]. Thresholds were applied to convert NOS scores into Agency for Healthcare Research and Quality (AHRQ) standards, categorizing studies as good, fair, or poor quality (Appendix B). This allowed for the standardized assessment of methodological quality, including non-randomized studies. Any disagreements between reviewers were resolved through discussion with a third author.

### 2.6. Data Synthesis

For the presentation of the included studies, the following syntheses were performed:

(1) Quantitative analysis: Studies reporting paternity rates, age at the time of treatment, and the use of ART were assessed. These outcomes were reported either collectively or stratified by unilateral and bilateral cryptorchidism, depending on the individual study. A meta-analysis was not conducted due to considerable heterogeneity across studies in terms of design, outcome definitions, and follow-up periods. Further statistical analyses from the included studies are presented in a separate table.
(2) Visual analysis: Studies reporting paternity rates as percentages were illustrated in a graph grouped by unilateral versus bilateral cryptorchidism. Furthermore, two graphs were constructed to illustrate paternity rates in relation to age at the time of treatment. In studies reporting age, either an interval or mean age was used for illustration. Finally, a graph was included to illustrate the use of ART among men treated for cryptorchidism during childhood.

## 3. Results

### 3.1. Study Selection

**Figure 1:**
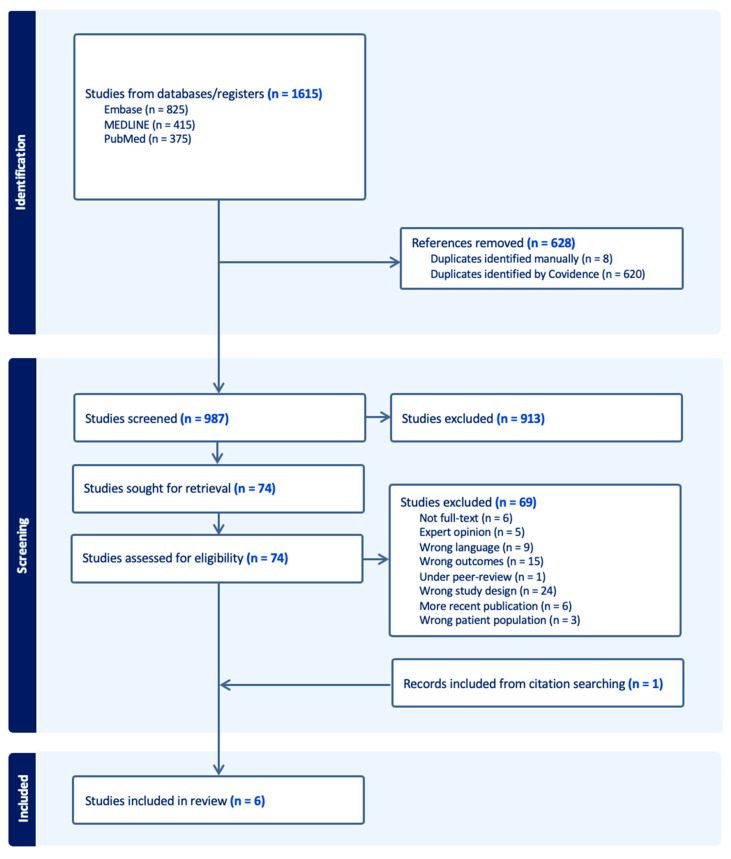
Flowchart outlining the selection of the included studies in the systematic review on the treatment of cryptorchidism in childhood.

The search strategy is presented in the PRISMA flowchart (Figure 1). A total of 1615 studies were identified through Embase, Medline, and PubMed. A total of 620 duplicates were identified by Covidence, and 8 duplicates were identified manually. Overall, 987 records were screened, with 913 records excluded based on their titles and abstracts. We excluded a total of 69 studies during full-text screening for various reasons: 24 records due to wrong study design, 15 records due to wrong outcome, 9 records due to wrong language, 6 records due to not being full texts, 6 records due to there being a more-recent publication, 5 records due to expert opinion, 3 records due to wrong patient populations, and 1 record due to being under peer review. Five studies met the eligibility criteria for this systematic review. During citation search, one record met the eligibility criteria except for the year of publication. A total of six studies were included for further analysis. 

The studies included in this review were conducted in Australia, Europe, and the United States of America. The largest study was conducted in Western Australia in 2018 and included a total of 350,835 men, with a sample of 7499 men who had a history of cryptorchidism; see Table 1. The majority of the studies focused on paternity outcomes among men diagnosed with an undescended testis in childhood who underwent orchiopexy. One study specifically examined infertile men who had been treated for cryptorchidism during childhood. Most participants were identified through medical records and subsequently surveyed, while one study assessed outcomes using record linkage. The age at treatment ranged from 1 month to 20 years, presented in Table 1 and Table 2. The reported paternity rate ranged from 14% to 89.7% among men treated for unilateral UDT and from 8% to 65.3% among men treated for bilateral UDT; see Table 1. Some of the studies accounted for the use of ART; see Table 1 and Table 2. For further statistical results, see Appendix C.

**Table 1:** Characteristics and outcomes of included studies in the systematic review.

Study ID	Design	Sample Size	UDT Group	Controls	Age Groups	Age at Treatment	Groups	Participants	Attempting Paternity	Paternity (%)	No Paternity (%)	Use of ART %	Other Outcomes
Schneuer et al. [13] 2018 Australia	Population-based cohort study	350,835	7499	341,000	<18 months	Mean age of UDT: 5.6 years ± 3.9 years	Unilateral	2765	-	519 (18.8)	- (-)	UDT: 0.9	Risk of testicular cancer, HR for paternity, and RR for the use of ART
					18 months–5 years		Bilateral	351	-	61 (17.4)	- (-)	-	
					≥6		Unaffected	239,239	-	49,298 (20.6)	- (-)	Unaffected: 0.3	
Van Brakel et al. [14] 2013 Netherlands	Long-term follow-up study	225	62	53	≤12 months	Median age of UDT: 3.0 years (0.1–14.6)	Unilateral	55	11	6 (55)	45 (-)	-	Fertility potential, including testis volume, hormone levels, and semen analysis
					≤18 months	Unilateral: 2.8 years (0.1–10.3)	Bilateral	7	-	- (-)	- (-)	-	
					≤24 months		Controls	53	29	25 (86)	14 (-)	-	
Paasch et al. [15] 2004 Germany	Clinical study	1648	167	374		Mean age of UDT: 6.8 ± 3.3 years	Unilateral	130	71	16 * (22 *)	78 (-)	IVF: 1, ICSI: 7	Semen analysis, hormone levels, and conception use
							Bilateral	37	26	4 * (16 *)	84 (-)	ICSI: 8	
							Controls	374	176	81 * (46.1 *)	53.9 (-)	IVF: 6.9, ICSI: 3.5, and IUI: 2.8	
Lee et al. [16] 1995 USA	Epidemiological study	-	363	336	<1, 1, 2, 3, 4, 5, 6, 7, 8, 9, 10, 11, 12, 13, 14, and 15 years	Unilateral interval: 1 month–15 years	Unilateral	243	203	183 ** (90.2)	9.8 (-)	-	Paternity among married or cohabitated men, conception related to time attempting paternity, age at orchiopexy among men attempting paternity within marriage or cohabitation, age at orchiopexy, and time of unprotected intercourse
					-	Bilateral interval: 1 month–13 years	Bilateral	38	31	20 ** (64.5)	35.5 (-)	-	
							Controls	267	218	203 ** (93.1)	6.9 (-)	-	
Lee et al. [17] 2001 USA	Cohort study	1405	-	708	-	-	Unilateral	609	359	322 (89.7)	10.3 (-)	-	Paternity in relation to sperm density, hormone levels, preoperative testis location and size, adult testis volume, and RR of infertility
					1.9, 2.7, 2.9, 3.2, 6.0, 7.4, 9.8, and 10.2 years	Bilateral interval: 1.9–10.2 years	Bilateral:	88	49	32 (65.3 ***)	34.7 (-)	-	
							Controls:	708	443	413 (93.2)	6.8 (-)	-	
Cendron et al. [18] 1989 USA	Critical long-term retrospective analysis	40	40	-	0–4 years	Mean age of UDT: 7 years	Unilateral	30	23	20 (87)	- (-)	-	Sperm count and paternity, fertility index and paternity
					5–9 years	Unilateral interval: 1–14 years	Bilateral	10	9	3 (33)	- (-)	-	
					10–15 years	Bilateral interval: 1–11 years	Controls	-	-	- (-)	- (-)	-	

Note. UDT: undescended testis, ART: assisted reproductive technology, HR: hazard ratio, RR: risk ratio, IVF: in vitro fertilization, ICSI: intracytoplasmic sperm injection, and IUI: intrauterine insemination. * Numbers summed: the total of spontaneous conception, conception after IVF, conception after IUI, and conception after ICSI [15]. ** Numbers summed: the total of 152 men (75%) with unilateral UDT, 14 men (45.2%) with bilateral UDT, and 178 men (81.6%) in the control group that attempted paternity for less than one year. A significant difference was observed between the bilateral group and the other groups in terms of the duration of regular intercourse without contraception prior to the conception of their first child [16]. *** Significantly lower than both unilateral UDT and the controls by the χ^2^-test. *p*-value < 0.001.

**Table 2:** Age groups, use of ART, and statistical significance of outcomes in included studies.

Study ID	Age Groups	Ages	Sample size	Type of Cryptorchidism	Paternity %	Use of ART %	Significance of Age at Orchiopexy
Schneuer et al. [13] 2018	Intervals	<18 months	1202	UDT	13.0	0.5	<18 months adjusted HR of 0.83 with 95% CI 0.70–0.98 for paternity
		18 months–5 years	3208	UDT	25.2	1.9	18 months to 5 years adjusted HR of 0.79 with 95% CI 0.71–0.87 for paternity
		6–20 years	3049	UDT	34.0	2.0	6 to 20 years adjusted HR of 0.78 with 95% CI 0.69–0.88 for paternity
	Mean age	5.6 ± 3.9 years		UDT	26.9	1.7	
Van Brakel et al. [14] 2013	Intervals	<12 months	8	*-*	*-*	*-*	*p*-value participation rate: 0.248
		<18 months	12	*-*	*-*	*-*	*p*-value participation rate: 0.176
		<24 months	24	*-*	*-*	*-*	*p*-value participation rate: 0.652
	Median age	3.0	62	UDT	*-*	*-*	
	Unilateral median age	2.8 years (0.1–10.3)	55	Unilateral	55	*-*	
Paasch et al. [15] 2004	Mean age	6.8 ± 3.3 years		Unilateral	22 *	IVF: 1 ICSI: 7	Significant level not reported
				Bilateral	16 *	ICSI: 8	
Lee et al., 1995 [16]	Age in years	<1, 1, 2, 3, 4, 5, 6, 7, 8, 9, 10, 11, 12, 13, 14, and 15	Paternity: 184; no paternity: 25	-	*-*	*-*	No significant difference
Lee et al., 2001 [17]	Age in years	1.9, 2.7, 2.9, 3.2, 6.0, 7.4, 9.8, and 10.2	8	Bilateral	60	-	Significant level not reported
Cendron et al., 1989 [18]	Intervals	0–4 years	5	Unilateral	80.0 **	*-*	Significant level not reported
			3	Bilateral	33.3 **	*-*	
		5–9 years	12	Unilateral	100 **	*-*	
			3	Bilateral	33.3 **	*-*	
		10–15 years	7	Unilateral	71.4 **		
			3	Bilateral	33.3 **		
	Mean age	7.0					

Note: ART: assisted reproductive technology, UDT: undescended testis, HR: hazard ratio, 95% CI: 95 percent confidence interval, IVF: in vitro fertilization, ICSI: intracytoplasmic sperm injection, CG: cryptorchidism group, and NCG: non-cryptorchidism group. * Numbers summed: The total of spontaneous conception, conception after IVF, conception after IUI, and conception after ICSI [15]. ** Numbers calculated from Figure 2. Age at surgery at paternity in Cendron et al. [18].

**Figure 2:**
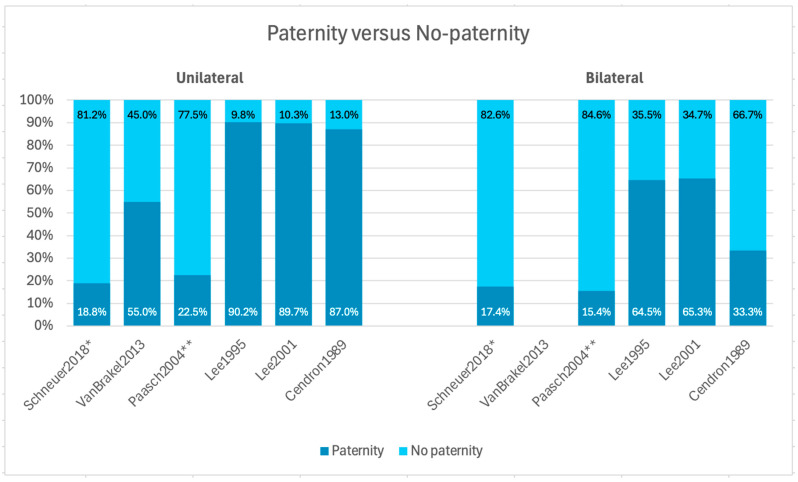
Paternity rates in unilateral vs. bilateral UDT compared to no paternity in the included studies [13,14,15,16,17,18]. The data is presented as percentages, illustrating the differences in the likelihood of achieving paternity between men treated for unilateral and bilateral UDT. Note: UDT: undescended testis, ☆: RoB good quality, * binary outcome: child or no child, and ** population: non-obstructive azoopermia (NOA) patients with cryptorchidism.

### 3.2. Level of Study Evidence

Table 3 provides an overview of the risk of bias assessment for the included studies, conducted using the Newcastle–Ottawa Scale (NOS). According to AHRQ criteria, the study of Schneuer et al. [13] was rated as having ‘good’ overall quality. Additionally, the studies of Lee et al. [16,17] were given four stars in the selection domain and two stars in the comparability domain. The study of Paasch et al. [15] was given three stars in the selection domain and one star in the comparability domain due to the lack of adjustment for age. The study of van Brakel et al. [14] was given two stars in selection due to missing descriptions regarding the ascertainment of exposure. The studies from Paasch et al. [15] and Cendron et al. [18] lacked external validity due to highly specific populations compared to the general population. The populations in both studies were highly specific, as they required participants with UDT and either biopsy or infertility, limiting their generalizability to the broader population [15,18].

In the outcome domain, all studies except for the study by Schneuer et al. [14,15,16,17,18] were rated as ‘poor’, primarily due to reliance on self-reported outcomes. 

A follow-up period of 40 years from birth was selected to ensure sufficient time for assessing paternity outcomes. In Denmark, the average age of first-time fathers was 31.9 years in 2024 [19]. In the United States, the average paternal age at the time of a child’s birth increased from 27.4 years in 1972 to 30.9 years in 2015 [20]. By extending the observation window to the age of 40, the study maximizes the probability that most individuals have reached an age by which they have had the opportunity to attempt paternity.

All six studies had follow-up durations of less than 31 years. Although some studies included a wide age range, the youngest participants did not meet the accepted follow-up criteria, which introduced a risk of bias. As follow-up was defined in this review as the time from birth to study participation, the studies of Lee et al. [16,17] and Cendron et al. [18] were considered not to meet the required duration. These studies reported only age at the time of treatment, not at participation, and since the men were younger than the defined follow-up period at the study’s initiation, it must be assumed that the criterion was not fulfilled.

In the two studies of Lee et al. [16,17], the adequacy of cohort follow-up is unclear, as only the number of participants is reported without specifying how many individuals were initially approached. Therefore, the follow-up rate remains undetermined, which increases the risk of bias. For a detailed risk of bias assessment, see Appendix B. 

### 3.3. Paternity Rate: Unilateral Versus Bilateral

**Figure 3:**
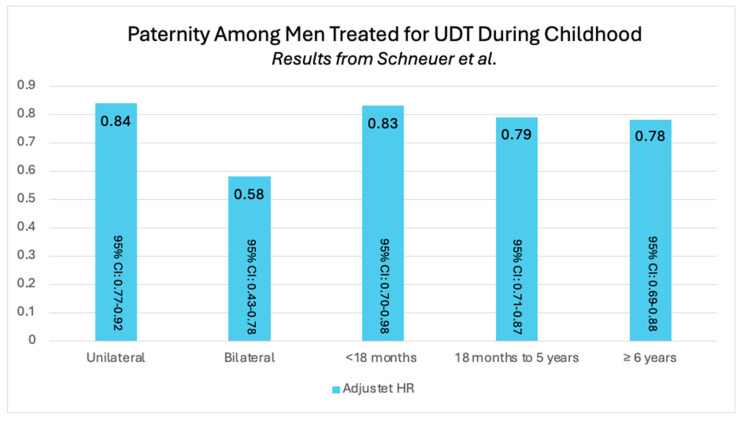
Adjusted hazard ratio (HR) for paternity among men born with UDT, stratified by unilateral and bilateral cases as well as by different age groups. The data illustrate how the likelihood of achieving paternity varies depending on the type of UDT and the age at treatment. Results from Schneuer et al. [13]. Note: UDT: undescended testis, 95% CI: 95% confidence interval.

The difference in paternity rates between unilateral versus bilateral UDT is illustrated in Figure 2. Results from five of the six studies indicate that the paternity rate is higher among men treated for unilateral UDT compared to men treated for bilateral UDT [13,15,16,17,18]. In the study by Schneuer et al. [13], paternity rates remained significantly lower compared to the unaffected reference group. The hazard ratio (HR) for paternity among men treated for unilateral cryptorchidism was 0.84 (95% confidence interval (95% CI): 0.77–0.92), while the HR for those treated for bilateral cryptorchidism was 0.58 (95% CI: 0.50–0.67), indicating a statistically significant reduction in paternity rates for both groups [13]. The HR is illustrated in Figure 3.

In contrast, the study by van Brakel et al. [14] found no significant difference in paternity rates between men with unilateral cryptorchidism and the control group, with a reported *p*-value of 0.8. 

The study by Paasch et al. [15] assessed conception rate as a proxy for paternity. The conception rate among infertile patients with bilateral cryptorchidism was not significantly lower compared to infertile patients with unilateral cryptorchidism, indicating no statistically significant difference between these two groups. However, the spontaneous conception rate in the infertile cryptorchidism group (CG) was significantly lower than in the infertile non-cryptorchidism group (NCG), with a reported *p*-value of <0.01. Furthermore, the total conception rate in the CG was significantly lower than in the NCG (*p* < 0.05), and the non-conception rate in the CG was also significantly lower than in the NCG (*p* < 0.01) [15].

The studies by Lee et al. [16,17], based on the same cohort, provide additional evidence regarding the impact of cryptorchidism on paternity. In their 1995 publication [16], no significant difference was observed in the proportion of married men with unilateral UDT who had fathered children compared to married controls. In contrast, a significant difference was found between men with bilateral UDT and married controls who had fathered children (*p* < 0.005). Additionally, among married men who had attempted to conceive, those with bilateral UDT had significantly lower paternity rates compared to the control group (*p* < 0.001) [16]. The follow-up publication by Lee et al. [17] in 2001 shows that among men who had attempted paternity, the paternity rate in the bilateral UDT group was significantly lower than in both the unilateral group and the control group (*p* < 0.001). Furthermore, the relative risk (RR) of infertility in the bilateral group was 5.3, with a highly significant *p*-value of <0.0001 [17].

One of the included studies did not report any levels of statistical significance for the outcomes presented [18].

### 3.4. Paternity Rate: Age of Treatment

**Figure 4:**
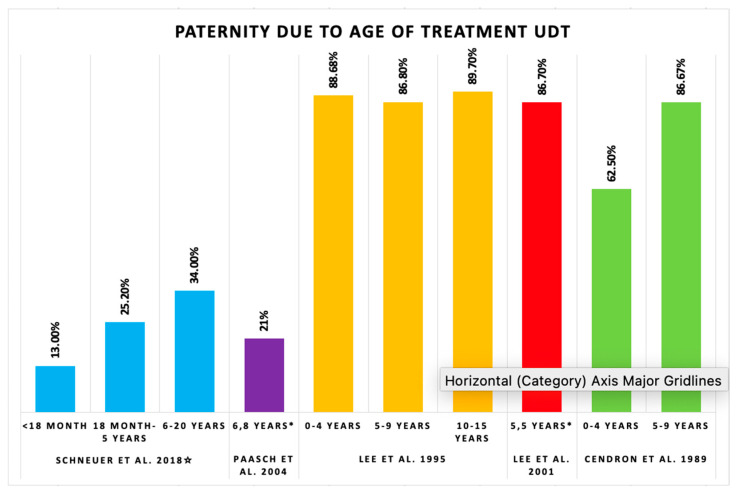
Percentage of men born with UDT who have achieved paternity, divided into different age groups in each of the included studies [13,15,16,17,18]. Note: UDT: undescended testis, ☆: RoB good quality, and *: mean age.

As illustrated in Figure 4, all studies except for the study of van Brakel et al. [13,15,16,17,18] assessed paternity outcomes in relation to age at treatment for undescended testes.

The population-based study by Schneuer et al. [13] reported the lowest paternity rates, with only 13.0% of individuals achieving paternity when treated before 18 months of age. A higher paternity rate of 25.2% was observed among those treated between 18 months and 5 years. Treatment between 6 and 20 years resulted in a paternity rate of 34%. Additionally, the study examined the hazard ratio (HR), by comparing the likelihood of paternity among men treated for cryptorchidism with the background population (reference group = 1). The highest HRs were observed among men who underwent orchiopexy before 18 months of age (adjusted HR: 0.83; 95% CI: 0.70–0.98), whereas the lowest HRs were found in those who were operated on at later age (18 months to 5 years: HR of 0.79; 95% CI: 0.71–0.87, and 6 and 20 years: HR of 0.78; 95% CI: 0.69–0.88), shown in Figure 3. Hazard ratios suggest significantly lower paternity rates in comparison to the unaffected reference group [13].

The study of van Brakel et al. [14] did not compare paternity rates by age groups but found no significant differences between the number of participants treated before 12, 18, or 24 months (*p* = 0.248, 0.176, and 0.652, respectively).

The study by Paasch et al. [15] observed a paternity rate of 21% at a mean treatment age of 6.8 years, without reported significance.

In contrast, Lee et al. [16,17] reported consistently high paternity rates across all age groups, with no statistically significant differences observed or reported.

Cendron et al. [18] found higher paternity rates among those treated between 5 and 9 years (86.7%) compared to those treated before the age of 5 (62.5%), though no statistical significance was reported.

### 3.5. Paternity Rate: Use of Assisted Reproductive Technology (ART)

**Figure 5:**
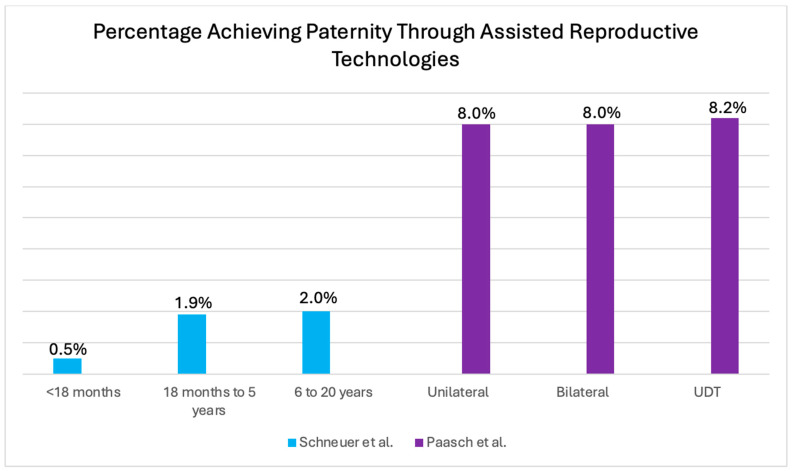
The figure shows the percentage of men who achieved paternity using ART, stratified by age at treatment (<18 months, 18 months to 5 years, and 6 to 20 years) based on data from Schneuer et al. [13] and by laterality of UDT (unilateral, bilateral, or overall UDT) based on data from Paasch et al. [15]. Note: UDT: undescended testis.

The studies of Schneuer et al. [13] and Paasch et al. [15] reported on the use of ART, illustrated in Figure 5.

The study of Schneuer et al. [13] found that delayed orchidopexy (>18 months) was associated with an increased need for ART. For every six-month delay of orchiopexy, the risk of the future use of ART increases by 5% and reduces the paternity rate by 1%. Additionally, boys operated on for UDT had more than twice the risk of needing ART compared to controls (adjusted RR = 2.26, CI: 1.58–3.25). When stratified by age at surgery, those treated before 18 months showed an adjusted RR of 1.33 (95% CI: 0.43–4.13), indicating no statistically significant increase in the likelihood of requiring ART. However, significantly higher risks were observed in those treated between 18 months and 5 years (adjusted RR = 2.74; 95% CI: 1.72–4.37) and between 6 and 20 years (adjusted RR = 2.16; 95% CI: 1.16–4.03) [13]. The results indicate that an early intervention reduces the need for ART.

In contrast, the study by Paasch et al. [15] found no significant difference in conception rates between unilateral and bilateral cryptorchidism, and this result was unaffected by ICSI treatment. In contrast, the *p*-value for conception after IVF in the CG was significantly lower than in the NCG (*p* < 0.05) [15].

## 4. Discussion

Across studies, men treated for bilateral cryptorchidism consistently showed lower paternity rates compared to those with unilateral undescended testes, whose fertility outcomes were often comparable to those of controls. Delayed orchiopexy did not appear to significantly impair fertility outcomes when surgery was performed during childhood. Early orchidopexy (<18 months) was associated with improved fertility and reduced ART use. This pattern was supported by a large population-based study, who reported an increased risk of ART use for every 6-month delay in surgery [13]. However, findings from smaller-cohort studies were more heterogeneous and less conclusive.

### 4.1. Explanations and Comparisons with Literature

To our knowledge, no previous systematic review has exclusively examined studies comparing paternity outcomes following unilateral versus bilateral cryptorchidism.

Our review found that the likelihood of paternity was lower in males with bilateral UDT compared to those with unilateral UDT. Rohayem et al. [21] found that men with a history of bilateral UDT had significantly reduced sperm concentrations compared to controls. In addition, reduced testicular volume is often observed in men with UDT. Reduced testicular volume has also been reported in this population and may further contribute to impaired spermatogenesis. Together, these factors may help explain the lower fertility potential observed in men with bilateral cryptorchidism [21].

In contrast, Lee et al. [16,17] found no significant difference in paternity rates between men with unilateral cryptorchidism and controls. Meanwhile, Bartoletti et al. [22] followed 192 patients for 16 years and observed improved semen quality in those treated with hormonal therapy, either alone or in combination with surgery, compared to surgery alone or no treatment. Notably, no differences were found between unilateral and bilateral cases in this cohort, suggesting the possibility of bilateral testicular involvement even in unilateral presentations [22]. Supporting this, a study by Hadziselimovic et al. [23] found that in patients with unilateral cryptorchidism, impaired transformation of Ad spermatogonia was observed in 70% of the contralateral, scrotal testes. Furthermore, sperm concentrations were strongly correlated with the number of Ad spermatogonia present at the time of orchidopexy (*p* < 0.001). These findings suggest that unilateral cryptorchidism may represent bilateral testicular dysfunction, where the subclinical impairment of the apparently unaffected testis could contribute to reduced overall fertility potential [23].

Furthermore, our review shows that delayed orchidopexy does not significantly affect fertility outcomes if the surgery is performed during childhood. Current clinical guidelines recommend that orchidopexy should be performed between 6 and 18 months of age, as endorsed by the American Academy of Pediatrics (AAP) Section on Urology, the European Association of Urology (EAU), the Nordic Consensus Statement, and the American Urological Association (AUA). Although postponing surgery may slightly increase the risk of testicular cancer, it does not seem to have a major impact on fertility if performed within the recommended age range [24].

A systematic review of Chan et al. [7] included a randomized controlled trial study looking at fertility potential, defined as testicular catch-up growth between 9 months and 4 years of age. Those who had orchiopexy at the age of 9 months had significant catch-up growth of the repaired testis compared to those who underwent orchiopexy at 3 years, who had no testicular growth before or after surgery. When looking at, e.g., sperm count and spermatozoa, Chan et al. [7]’s highest-quality studies showed that fertility potential was highest when orchiopexy was performed before the age of one year, as this timing was associated with significantly higher sperm counts and a greater proportion of highly motile spermatozoa compared to surgery performed between the ages of one and two years. The consensus in the study of Chan et al. [7] was that earlier orchiopexy had better outcomes [7]. 

Similarly, Rohayem et al. [21] reported that, in men with UDT, the age at the correction of UDT was inversely correlated with both testicular volume and sperm concentration in semen, supporting the importance of early intervention [21].

In our highest-quality included study, earlier orchidopexy was associated with higher paternity rates, as indicated by the reported hazard ratios [13]. However, the two other studies found higher absolute paternity rates among individuals treated later in childhood [16,18]. A plausible explanation for this unexpected finding could be selection bias. Thus, the most severely affected cases might mainly have been treated in early life, while cases with retractile testicles or testicles with a more-favorable localization might have been overrepresented in boys treated in late childhood [25]. Notably, none of the included studies explicitly reported excluding boys with retractile testicles from their cohorts.

Many patients with a history of cryptorchidism have reduced semen quality, or even azoospermia. A history of cryptorchidism, unilateral or bilateral, was found in more than one-quarter (98 out of 328) of an unselected cohort of men with azoospermia [26]. However, since men with cryptorchidism usually have a very heterogeneous testicular histological pattern [27], it is almost always possible to find foci with sperm production with testicular sperm extraction (TESE) or microsurgical testicular sperm extraction (micro-TESE). This suggests that, even in the absence of ejaculated sperm, many men with a history of cryptorchidism still have the potential to father biological children through ART.

These results suggest that while early treatment is preferable, delayed treatment still leads to lower paternity rates but may not drastically reduce fertility outcomes, particularly if correction is performed in early childhood. Studies by Paasch et al. [15], Lee et al. [16,17], and Cendron et al. [18] found no significant differences in paternity rates across age groups, supporting the finding that delayed treatment may have less impact on fertility than often assumed.

### 4.2. Strengths and Limitations

This review demonstrates several methodological strengths. Most notably, the literature search was comprehensive and systematic, covering three databases and using broad search terms to ensure the inclusion of all relevant published studies, thereby minimizing selection bias and increasing the robustness of the evidence base. 

A further strength lies in the use of paternity as a primary outcome. Though paternity is self-reported, it is a straightforward and well-defined outcome, reducing the risk of subjective interpretation or reporting bias compared to more-sensitive measures such as infertility or conception attempts. While not validated through medical or population registers, its clarity enhances its reliability as an outcome measure. This strengthens the internal validity of the results and supports its use in studies on male reproductive health.

In addition to the general methodological strengths, several notable strengths were identified within the individual included studies. A few of the included studies, such as Schneuer et al. [13] and Lee et al. [16,17], featured large sample sizes and appropriate control groups, often drawn from the same source population, which enhances internal comparability and external validity. Although all included studies were retrospective in design, exposure data, such as age at orchiopexy and diagnosis of UDT, were typically extracted from medical records, thereby minimizing the risk of misclassification. One study utilized record linkage, allowing for an exceptionally high follow-up rate of 98.7% [13]. In contrast, the remaining studies relied on self-reported outcomes [14,15,16,17,18].

Despite these strengths, some limitations must be acknowledged. The overall level of evidence in our study is inherently limited, as all included studies were non-randomized observational studies, primarily retrospective due to ethical constraints. This increases the potential for recall bias. 

There is also uncertainty regarding confounding by indication, as boys with UDT may have a higher baseline risk of androgen-related complications, independent of surgical intervention, as suggested by Ruili Li et al. [28]. This intrinsic biological factor may confound the association between timing of surgery and later fertility. 

A notable limitation of the included studies [13,14,15,16,17,18] is the lack of genetic verification through DNA testing to confirm biological paternity. Incorporating such testing would strengthen the validity of paternity outcomes and reduce potential bias.

When assessing paternity as the primary outcome, it is essential to acknowledge that female fertility significantly influences a couple’s ability to conceive. Globally, infertility affects an estimated 8% to 12% of couples of reproductive age, with female-related factors accounting for approximately 30% of these cases. A major contributor to female infertility is secondary infertility resulting from reproductive tract infections [29]. The lack of information on female fertility status in the included studies represents a notable limitation, as it impedes a comprehensive interpretation of the paternity outcomes [13,14,15,16,17,18].

Finally, substantial heterogeneity was observed across study designs, intervention timing, and outcome definitions, making direct comparisons difficult and possibly contributing to variability in reported effects. This limits our ability to conduct a meta-analysis. In some cases, small sample sizes and limited long-term follow-up further restrict the interpretability and generalizability of the findings.

The included studies exhibited several methodological limitations. Notably, two studies included highly selected populations, focusing on subgroups of men with a history of both cryptorchidism and infertility or cryptorchidism and testicular biopsy [15,18], reducing the generalizability of their findings. These cohorts are not representative of the broader population of men with cryptorchidism and may overestimate fertility impairment.

Time-related and attrition biases also pose a risk. Studies varied considerably in their follow-up rates, with high attrition reported in some (e.g., 72.4% in van Brakel et al. [14], 49.5% in Paasch et al. [15]), while others did not report follow-up data at all [16,17]. Such variation may lead to selection bias, especially if individuals who achieved paternity were more likely to respond. Additionally, Schneuer et al. [13] assessed only actual paternity and did not account for attempts to conceive, introducing a potential risk of immortal time bias. Though this may have been mitigated by similar likelihoods of attempting conception in exposed and unexposed groups.

### 4.3. Perspectives

This review supports current guidelines recommending early orchidopexy, ideally before 18 months, to optimize fertility, especially in bilateral cases. While delayed surgery may still result in acceptable outcomes, early intervention remains preferable. Future studies should include long-term follow-up and investigate the role of hormonal therapy, which is underreported in the current literature. From a public health perspective, improved early detection and timely referral systems are essential to support fertility outcomes later in life. In particular, registry-based studies linking surgical timing with reproductive outcomes could provide valuable evidence for both clinical care and policy development. There is a clear need for high-quality, well-designed studies to strengthen the evidence base and guide future clinical recommendations on the optimal timing of treatment for cryptorchidism, with the aim of improving fertility outcomes and reducing the need for ART.

## 5. Conclusions

Men treated for bilateral cryptorchidism had consistently lower paternity rates compared to those with unilateral involvement, whose fertility outcomes often resembled those of the general population. Early orchidopexy, ideally before 18 months, was associated with improved fertility outcomes and the reduced use of ART. Although some studies paradoxically reported higher paternity rates with later surgery, this may be attributed to selection bias and smaller cohorts. Overall, the evidence supports current clinical guidelines recommending early surgical intervention, given its association with improved fertility outcomes, increased testicular volume, higher sperm concentration, reduced need for ART, and a lower risk of malignancy. This review highlights the need for further research on long-term fertility outcomes. In particular, the potential role of hormonal therapy remains insufficiently studied and therefore merits further investigation.

## Figures and Tables

**Table 3:** Overview of risk of bias assessment of the included studies.

	Newcastle–Ottawa Quality Assessment Scale									Score
Included Studies	1	2	3	4	5		6	7	8	Total Number of Stars
Schneuer et al. [13]	a ☆	a ☆	a ☆	a ☆	a☆	b ☆	b ☆	b	b ☆	8
Van Brakel et al. [14]	b ☆	b	d	a ☆	a☆	b ☆	c	b	c	4
Paasch et al. [15]	c	a ☆	a ☆	a ☆	-	b ☆	c	b	c	4
Lee et al. [16]	b ☆	a ☆	a ☆	a ☆	a☆	b ☆	c	b	d	6
Lee et al. [17]	b ☆	a ☆	a ☆	a ☆	a☆	b ☆	c	b	d	6
Cendron et al. [18]	c	-	a ☆	a ☆	a ☆	b ☆	c	b	b☆	5

Note: Colors in the Newcastle-Ottawa Scale denote the study quality; green: high quality, orange: moderate quality, yellow: some concerns, red: low quality, ☆: represents a met quality criterion.

## Data Availability

Not applicable.

## Appendix A

**Table A1:** Final Embase search.

Final Search, Embase Classic + Embase < 1947 to 2025 6 February 2025 > 7 February 2025		
#	Searches	Results
1	cryptorchism/	17,914
2	Cryptochi*.mp. [mp=title, abstract, heading word, drug trade name, original title, device manufacturer, drug manufacturer, device trade name, keyword heading word, floating subheading word, candidate term word]	160
3	Undescended test*.mp. [mp=title, abstract, heading word, drug trade name, original title, device manufacturer, drug manufacturer, device trade name, keyword heading word, floating subheading word, candidate term word]	4822
4	Maldescensus testis.mp. [mp=title, abstract, heading word, drug trade name, original title, device manufacturer, drug manufacturer, device trade name, keyword heading word, floating subheading word, candidate term word]	94
5	Testis, undescended.mp. [mp=title, abstract, heading word, drug trade name, original title, device manufacturer, drug manufacturer, device trade name, keyword heading word, floating subheading word, candidate term word]	16
6	1 or 2 or 3 or 4 or 5	18,784
7	orchidopexy/	4836
8	Orchidopexi*.mp. [mp=title, abstract, heading word, drug trade name, original title, device manufacturer, drug manufacturer, device trade name, keyword heading word, floating subheading word, candidate term word]	252
9	Orchiopex*.mp. [mp=title, abstract, heading word, drug trade name, original title, device manufacturer, drug manufacturer, device trade name, keyword heading word, floating subheading word, candidate term word]	1998
10	surgery/	1,027,013
11	gonadorelin/	42,565
12	gonadotropin releasing*.mp. [mp=title, abstract, heading word, drug trade name, original title, device manufacturer, drug manufacturer, device trade name, keyword heading word, floating subheading word, candidate term word]	20,815
13	gonadorelin.mp. [mp=title, abstract, heading word, drug trade name, original title, device manufacturer, drug manufacturer, device trade name, keyword heading word, floating subheading word, candidate term word]	70,900
14	GnRh*.mp. [mp=title, abstract, heading word, drug trade name, original title, device manufacturer, drug manufacturer, device trade name, keyword heading word, floating subheading word, candidate term word]	37,271
15	Hcg.mp. [mp=title, abstract, heading word, drug trade name, original title, device manufacturer, drug manufacturer, device trade name, keyword heading word, floating subheading word, candidate term word]	41,969
16	Surger*.mp. [mp=title, abstract, heading word, drug trade name, original title, device manufacturer, drug manufacturer, device trade name, keyword heading word, floating subheading word, candidate term word]	4,998,509
17	7 or 8 or 9 or 10 or 11 or 12 or 13 or 14 or 15 or 16	5,100,952
18	paternity/	4904
19	fertility/	79,471
20	sperm quality/	10,228
21	Paternit*.mp. [mp=title, abstract, heading word, drug trade name, original title, device manufacturer, drug manufacturer, device trade name, keyword heading word, floating subheading word, candidate term word]	9970
22	Fertilit*.mp. [mp=title, abstract, heading word, drug trade name, original title, device manufacturer, drug manufacturer, device trade name, keyword heading word, floating subheading word, candidate term word]	186,727
23	Sperm quality.mp. [mp=title, abstract, heading word, drug trade name, original title, device manufacturer, drug manufacturer, device trade name, keyword heading word, floating subheading word, candidate term word]	14,714
24	Semen quality.mp. [mp=title, abstract, heading word, drug trade name, original title, device manufacturer, drug manufacturer, device trade name, keyword heading word, floating subheading word, candidate term word]	8610
25	live birth/	40,609
26	Live birth.mp. [mp=title, abstract, heading word, drug trade name, original title, device manufacturer, drug manufacturer, device trade name, keyword heading word, floating subheading word, candidate term word]	46,728
27	18 or 19 or 20 or 21 or 22 or 23 or 24 or 25 or 26	246,582
28	6 and 17 and 27	1012
29	28 and 1990: 2025.(sa_year).	825

Note: *: Terms are truncated by * at the root to capture all variations and suffixes.

**Table A2:** Final Ovid MEDLINE search.

Final Search, Ovid MEDLINE(R) ALL < 1946 to 6 February 2025 >, 7 February 2025		
Search Strategy:		
#	Searches	Results
1	Cryptorchism/	8788
2	Cryptorchi*.mp. [mp=title, book title, abstract, original title, name of substance word, subject heading word, floating sub-heading word, keyword heading word, organism supplementary concept word, protocol supplementary concept word, rare disease supplementary concept word, unique identifier, synonyms, population supplementary concept word, anatomy supplementary concept word]	11,809
3	Undescended test*.mp. [mp=title, book title, abstract, original title, name of substance word, subject heading word, floating sub-heading word, keyword heading word, organism supplementary concept word, protocol supplementary concept word, rare disease supplementary concept word, unique identifier, synonyms, population supplementary concept word, anatomy supplementary concept word]	3327
4	maldescensus testis.mp. [mp=title, book title, abstract, original title, name of substance word, subject heading word, floating sub-heading word, keyword heading word, organism supplementary concept word, protocol supplementary concept word, rare disease supplementary concept word, unique identifier, synonyms, population supplementary concept word, anatomy supplementary concept word]	54
5	testis, undescended.mp. [mp=title, book title, abstract, original title, name of substance word, subject heading word, floating sub-heading word, keyword heading word, organism supplementary concept word, protocol supplementary concept word, rare disease supplementary concept word, unique identifier, synonyms, population supplementary concept word, anatomy supplementary concept word]	22
6	1 or 2 or 3 or 4 or 5	12,751
7	orchidopexy/	711
8	orchidopexi*.mp. [mp=title, book title, abstract, original title, name of substance word, subject heading word, floating sub-heading word, keyword heading word, organism supplementary concept word, protocol supplementary concept word, rare disease supplementary concept word, unique identifier, synonyms, population supplementary concept word, anatomy supplementary concept word]	181
9	Orchiopex*.mp. [mp=title, book title, abstract, original title, name of substance word, subject heading word, floating sub-heading word, keyword heading word, organism supplementary concept word, protocol supplementary concept word, rare disease supplementary concept word, unique identifier, synonyms, population supplementary concept word, anatomy supplementary concept word]	1870
10	General Surgery/	41,328
11	Surger*.mp. [mp=title, book title, abstract, original title, name of substance word, subject heading word, floating sub-heading word, keyword heading word, organism supplementary concept word, protocol supplementary concept word, rare disease supplementary concept word, unique identifier, synonyms, population supplementary concept word, anatomy supplementary concept word]	3,278,595
12	gonadorelin/	30,319
13	gonadotropin releasing*.mp. [mp=title, book title, abstract, original title, name of substance word, subject heading word, floating sub-heading word, keyword heading word, organism supplementary concept word, protocol supplementary concept word, rare disease supplementary concept word, unique identifier, synonyms, population supplementary concept word, anatomy supplementary concept word]	37,791
14	gonadorelin.mp. [mp=title, book title, abstract, original title, name of substance word, subject heading word, floating sub-heading word, keyword heading word, organism supplementary concept word, protocol supplementary concept word, rare disease supplementary concept word, unique identifier, synonyms, population supplementary concept word, anatomy supplementary concept word]	290
15	GnRH*.mp. [mp=title, book title, abstract, original title, name of substance word, subject heading word, floating sub-heading word, keyword heading word, organism supplementary concept word, protocol supplementary concept word, rare disease supplementary concept word, unique identifier, synonyms, population supplementary concept word, anatomy supplementary concept word]	27,327
16	Hcg.mp. [mp=title, book title, abstract, original title, name of substance word, subject heading word, floating sub-heading word, keyword heading word, organism supplementary concept word, protocol supplementary concept word, rare disease supplementary concept word, unique identifier, synonyms, population supplementary concept word, anatomy supplementary concept word]	27,902
17	7 or 8 or 9 or 10 or 11 or 12 or 13 or 14 or 15 or 16	3,344,095
18	Paternity/	3399
19	Fertility/	46,463
20	Semen Analysis/	7064
21	paternit*.mp. [mp=title, book title, abstract, original title, name of substance word, subject heading word, floating sub-heading word, keyword heading word, organism supplementary concept word, protocol supplementary concept word, rare disease supplementary concept word, unique identifier, synonyms, population supplementary concept word, anatomy supplementary concept word]	7809
22	Fertilit*.mp. [mp=title, book title, abstract, original title, name of substance word, subject heading word, floating sub-heading word, keyword heading word, organism supplementary concept word, protocol supplementary concept word, rare disease supplementary concept word, unique identifier, synonyms, population supplementary concept word, anatomy supplementary concept word]	137,169
23	Sperm quality.mp. [mp=title, book title, abstract, original title, name of substance word, subject heading word, floating sub-heading word, keyword heading word, organism supplementary concept word, protocol supplementary concept word, rare disease supplementary concept word, unique identifier, synonyms, population supplementary concept word, anatomy supplementary concept word]	6773
24	semen quality.mp. [mp=title, book title, abstract, original title, name of substance word, subject heading word, floating sub-heading word, keyword heading word, organism supplementary concept word, protocol supplementary concept word, rare disease supplementary concept word, unique identifier, synonyms, population supplementary concept word, anatomy supplementary concept word]	6666
25	Live Birth/	6081
26	Live birth.mp. [mp=title, book title, abstract, original title, name of substance word, subject heading word, floating sub-heading word, keyword heading word, organism supplementary concept word, protocol supplementary concept word, rare disease supplementary concept word, unique identifier, synonyms, population supplementary concept word, anatomy supplementary concept word]	18,077
27	18 or 19 or 20 or 21 or 22 or 23 or 24 or 25 or 26	168,778
28	6 and 17 and 27	567

Note: *: Terms are truncated by * at the root to capture all variations and suffixes.

**Table A3:** Final PubMed search.

Final Search, PubMed, 7 February 2025		
Search Strategy:		
#	Searches	Results
7	(((“Cryptorchidism”[Mesh] OR cryptorchi* OR “undescended test*” OR “undescended testes” OR “maldescensus testis” OR “Testis Undescended”)) AND ((“Orchiopexy”[Mesh] OR Orchidopexi* OR “surgical procedures, operative”[Mesh] OR Surgery OR “Gonadotropin-Releasing Hormone”[Mesh] OR “Gonadotropin releasing*” OR Gonadorelin OR GnRH* OR hCG))) AND ((“Paternity”[Mesh] OR “Fertility”[Mesh] OR “Semen analysis”[Mesh] OR “Live birth”[Mesh] OR Paternit* OR Fertilit* OR “Sperm quality” OR “Semen quality” OR “Live Birth”)) Filters: Danish, English, Norwegian, Swedish, Humans from 1990–2025	375
6	(((“Cryptorchidism”[Mesh] OR cryptorchi* OR “undescended test*” OR “undescended testes” OR “maldescensus testis” OR “Testis Undescended”)) AND ((“Orchiopexy”[Mesh] OR Orchidopexi* OR “surgical procedures, operative”[Mesh] OR Surgery OR “Gonadotropin-Releasing Hormone”[Mesh] OR “Gonadotropin releasing*” OR Gonadorelin OR GnRH* OR hCG))) AND ((“Paternity”[Mesh] OR “Fertility”[Mesh] OR “Semen analysis”[Mesh] OR “Live birth”[Mesh] OR Paternit* OR Fertilit* OR “Sperm quality” OR “Semen quality” OR “Live Birth”)) Filters: Danish, English, Norwegian, Swedish, from 1990–2025	490
5	(((“Cryptorchidism”[Mesh] OR cryptorchi* OR “undescended test*” OR “undescended testes” OR “maldescensus testis” OR “Testis Undescended”)) AND ((“Orchiopexy”[Mesh] OR Orchidopexi* OR “surgical procedures, operative”[Mesh] OR Surgery OR “Gonadotropin-Releasing Hormone”[Mesh] OR “Gonadotropin releasing*” OR Gonadorelin OR GnRH* OR hCG))) AND ((“Paternity”[Mesh] OR “Fertility”[Mesh] OR “Semen analysis”[Mesh] OR “Live birth”[Mesh] OR Paternit* OR Fertilit* OR “Sperm quality” OR “Semen quality” OR “Live Birth”)) Filters: Danish, English, Norwegian, from 1990–2025	490
4	(((“Cryptorchidism”[Mesh] OR cryptorchi* OR “undescended test*” OR “undescended testes” OR “maldescensus testis” OR “Testis Undescended”)) AND ((“Orchiopexy”[Mesh] OR Orchidopexi* OR “surgical procedures, operative”[Mesh] OR Surgery OR “Gonadotropin-Releasing Hormone”[Mesh] OR “Gonadotropin releasing*” OR Gonadorelin OR GnRH* OR hCG))) AND ((“Paternity”[Mesh] OR “Fertility”[Mesh] OR “Semen analysis”[Mesh] OR “Live birth”[Mesh] OR Paternit* OR Fertilit* OR “Sperm quality” OR “Semen quality” OR “Live Birth”)) Filters: Danish, English, from 1990–2025	490
3	(((“Cryptorchidism”[Mesh] OR cryptorchi* OR “undescended test*” OR “undescended testes” OR “maldescensus testis” OR “Testis Undescended”)) AND ((“Orchiopexy”[Mesh] OR Orchidopexi* OR “surgical procedures, operative”[Mesh] OR Surgery OR “Gonadotropin-Releasing Hormone”[Mesh] OR “Gonadotropin releasing*” OR Gonadorelin OR GnRH* OR hCG))) AND ((“Paternity”[Mesh] OR “Fertility”[Mesh] OR “Semen analysis”[Mesh] OR “Live birth”[Mesh] OR Paternit* OR Fertilit* OR “Sperm quality” OR “Semen quality” OR “Live Birth”)) Filters: English, from 1990–2025	490
2	(((“Cryptorchidism”[Mesh] OR cryptorchi* OR “undescended test*” OR “undescended testes” OR “maldescensus testis” OR “Testis Undescended”)) AND ((“Orchiopexy”[Mesh] OR Orchidopexi* OR “surgical procedures, operative”[Mesh] OR Surgery OR “Gonadotropin-Releasing Hormone”[Mesh] OR “Gonadotropin releasing*” OR Gonadorelin OR GnRH* OR hCG))) AND ((“Paternity”[Mesh] OR “Fertility”[Mesh] OR “Semen analysis”[Mesh] OR “Live birth”[Mesh] OR Paternit* OR Fertilit* OR “Sperm quality” OR “Semen quality” OR “Live Birth”)) Filters: from 1990–2025	550
1	(((“Cryptorchidism”[Mesh] OR cryptorchi* OR “undescended test*” OR “undescended testes” OR “maldescensus testis” OR “Testis Undescended”)) AND ((“Orchiopexy”[Mesh] OR Orchidopexi* OR “surgical procedures, operative”[Mesh] OR Surgery OR “Gonadotropin-Releasing Hormone”[Mesh] OR “Gonadotropin releasing*” OR Gonadorelin OR GnRH* OR hCG))) AND ((“Paternity”[Mesh] OR “Fertility”[Mesh] OR “Semen analysis”[Mesh] OR “Live birth”[Mesh] OR Paternit* OR Fertilit* OR “Sperm quality” OR “Semen quality” OR “Live Birth”))	759

Note: *: Terms are truncated by * at the root to capture all variations and suffixes.

## Appendix B

**Table A4:** A detailed assessment of the risk of bias in the studies included in this systematic review.

	Selection				Comparability		Outcome			
Study ID	1.1	1.2	1.3	1.4	2.a	2.b	3.1	3.2	3.3	AHRQ
Schneuer et al. [13]	All liveborn boys with UDT in Western Australia	All liveborn boys in Western Australia	Hospital data or WARDA	Outcome not demonstrated at the baseline	Unilateral and bilateral UDTs were analyzed separately, but age was assessed for both groups combined	Adjusted for maternal age, birth weight, socioeconomic disadvantage, and hospital stay in first 15 years	Record linkage	Born in 1970 to 1999 and followed until 31 December 2016	The loss of 4726 participants (1.33%) occurred from the total of 353,336	Selection: 4 ☆ Comparability: 2 ☆ Outcome: 2 ☆ Overall: good
Van Brakel et al. [14]	The study includes men operated on before age of 2 and boys from a previous placebo-controlled trial	Control group from another study	No description	Outcome not demonstrated at the baseline	Age at orchiopexy and UDT laterality did not significantly differ between participants and non-participants	Adjusts for background characteristics in the unilateral UDT and control groups	Self-reported	UDT age: 20.1–37.9 years Control age: 23.0–49.8 years	A loss of 163 participants (72.4%) occurred from the total of 225	Selection: 2 ☆ Comparability: 2 ☆ Outcome: 0 ☆ Overall: poor
Paasch et al. [15]	UDT group with infertility	Infertile men attempting paternity without UDT	Winsperm 2000 database	Outcome not demonstrated at the baseline	Correlates for unilateral and bilateral UDTs, reporting only the mean age	Adjusts for use of fertility treatment	Self-reported	Age of 19–62 years	A loss of 268 participants (49.5%) occurred from a total of 541	Selection: 3 ☆ Comparability: 2 ☆ Outcome:0 ☆ Overall: poor
Lee et al. [16]	Men who had orchiopexy at the Children’s Hospital of Pittsburgh (1955–1969)	Controls matched on age, hospital, and surgery date for an unrelated minor problem	Medical records	Outcome not demonstrated at the baseline	Unilateral and bilateral UDTs were analyzed separately, but age was assessed for both groups combined	Adjusts for men attempting paternity, marital status, and time to conceive	Self-reported	Follow-up after orchiopexy is 26 to 40 years. No age of participants at participation	No statement	Selection: 4 ☆ Comparability: 2 ☆ Outcome: 0 ☆ Overall: poor
Lee et al. [17]	Men who had orchiopexy at the Children’s Hospital of Pittsburgh (1955–1975)	Controls matched on age, hospital, and surgery date for an unrelated minor problem	Medical records	Outcome not demonstrated at the baseline	Unilateral and bilateral UDTs were analyzed separately, but age was assessed for bilateral UDT	Adjusts for men attempting paternity	Self-reported	Follow-up after orchiopexy is 26 to 46 years. No age of participants at participation	No statement	Selection: 4 ☆ Comparability: 2 ☆ Outcome: 0 ☆ Overall: poor
Cendron et al. [18]	Men with UDT and orchiopexy with biopsy at the Children’s Hospital of Philadelphia	None	Hospital records	Outcome not demonstrated at the baseline	Unilateral and bilateral UDTs and age were analyzed separately	Adjusts for attempting paternity	Self-reported	Follow-up after biopsy is 30 to 40 years. No age of participants at participation	A loss of 9 participants (18.4%) occurred from a total of 49	Selection: 2 ☆ Comparability: 2 ☆ Outcome: 1 ☆ Overall: poor

Note: 1.1–1.4: selection questions according to the Newcastle–Ottawa Scale. 2.a and 2.b: comparability questions according to the Newcastle–Ottawa Scale. 3.1–3.3: outcome questions according to the Newcastle–Ottawa Scale. AHRQ: Thresholds for converting the Newcastle–Ottawa Scale to AHRQ standards (good, fair, and poor). Good quality: 3 or 4 stars in the selection domain AND 1 or 2 stars in the comparability domain AND 2 or 3 stars in the outcome/exposure domain; fair quality: 2 stars in the selection domain AND 1 or 2 stars in the comparability domain AND 2 or 3 stars in the outcome/exposure domain; and poor quality: 0 or 1 star in the selection domain OR 0 stars in the comparability domain OR 0 or 1 stars in the outcome/exposure domain. UDT: undescended testis.

## Appendix C

**Table A5:** Further statistical results from the included studies in this systematic review.

Study	Additionally Statistical Results
Schneuer et al. [13]	Unilateral UDT adjusted HR of 0.84 with 95% CI: 0.77–0.92 for paternity
	Bilateral UDT adjusted HR of 0.58 with 95% CI: 0.43–0.78 for paternity
	Paternity rates are lower in men with undescended testes, and delayed orchidopexy (>18 months) increases ART use
	A 6-month delay in orchidopexy raises ART risk by 5% and reduces paternity by 1%
	UDT had a two-fold increase in the risk of the future use of ART (adjusted RR of 2.26, 95% CI: 1.58–3.25)
	<18-month adjusted RR of 1.33 with 95% CI: 0.43–4.13 for the use of ART
	18 months to 5 years adjusted RR of 2.74 with 95% CI: 1.72–4.37 for the use of ART
	6 to 20 years adjusted RR of 2.16 with 95% CI: 1.16–4.03 for the use of ART
Van Brakel et al. [14]	*p*-value for the comparison of paternity rates between unilateral UDT participants and controls: 0.08
Paasch et al. [15]	The conception rate for patients with bilateral cryptorchidism was not significantly lower than the conception rate for patients with unilateral cryptorchidism
	*p*-value for spontaneous conception rate in the CG was significantly lower than in the NCG: <0.01
	*p*-value for total conception rate in the CG was significantly lower than in the NCG: <0.05
	*p*-value for non-conception rate in the CG was significantly lower than in the NCG: <0.01
	*p*-value for conception after IVF in the CG was significantly higher than in the NCG: <0.05
Lee et al. [16]	No significant difference between the proportion of unilateral UDT and control married men who had fathered children
	Significant difference between the bilateral UDT group with children compared to married controls with children. *p*-value < 0.005
	Significant difference between men with bilateral UDT who were married and had attempted compared to the control group. *p*-value < 0.001
Lee et al. [17]	Rates of paternity for those who have attempted paternity show a significantly lower rate for the bilateral group than for both the unilateral (*p*-value < 0.001) and control (*p*-value < 0.001) groups
	The RR for infertility is 5.3 in the bilateral group, with a *p*-value < 0.0001
Cendron et al. [18]	Statistical results not reported

Note. UDT: undescended testis, HR: hazard ratio, 95% CI: 95% confidence interval, ART: assisted reproductive technology, RR: risk ratio, CG: cryptochid group, NCG: non-cryptorchid group, and IVF: in vitro fertilization.
